# History of a Case of Lumbar Abscess Attended with Preternatural Anus in the Groin

**Published:** 1821-06

**Authors:** W. E. Horner

**Affiliations:** Demonstrator of Anatomy in the University of Pennsylvania.


### *History of a Case of Lumbar Abscess attended with Preternatural Anus in the Groin.* By W. E. Horner, M.D. Demonstrator of Anatomy in the University of Pennsylvania.

ON the 15th of August last, I was requested by Dr. Gebhard to examine a patient of his, who had died in Callowhill, near Garden-street, in consequence of psoas abscess. The account of the case afforded me was as follows:

James Culberton, a carter, aged twenty-four years, of a very strong and athletic constitution, in consequence of some injury or strain received in the prosecution of his business in August 1814, felt a severe pain arise in the lumbar and iliac regions, which was followed with fever and the common symptoms of inflammation.

Bleeding, purging, and other means usually resorted to on such occasions, were adopted, without any other effect than that of alleviating in some measure the intensity of the attack. From the inadequacy of the remedies to produce discussion, suppuration took place: the matter forming passed out under Poupart's ligament, and produced a prominent and fluctuating tumor, a little below it. This tumor was opened with a lancet, and a considerable quantity of pus discharged. With little subsequent attention the wound got well, and the energies of the patient's constitution seemed to have restored him in a short time to his ordinary state of health.

In the middle of the next winter, from being exposed to a snow-storm, he caught cold: the place in the groin which had been occupied by the tumor, and in the vicinity of the incision, became heated, painful, red, and tumefied; suppuration followed, and a spontaneous opening was made, through which the pus, mixed with feculent matter, was discharged. Doubt might have been entertained at this time in regard to the real nature of the feculent discharge; but the patient having eaten some rice shortly before, it so happened that a few grains of it passed undigested through the opening. This accident demonstrated that a connexion existed between the intestinal canal and the orifice in the thigh. A number of perplexing considerations grew out of this circumstance. 1st. It was thought that a femoral hernia, lying under the tumor, might have been wounded by the plunge of the lancet; but, upon a rigid scrutiny into the case, it appeared that the patient had never in his life been affected with any of its symptoms, and a local examination of the part itself at the time indicated nothing of the kind. It, however, was considered possible for it to have had a temporary existence, and to have been afterwards returned by the voluntary action of the bowels, assisted by the position of the patient. 2d. If the primitive inflammation in the lumbar and right iliac regions had invaded the intestinal canal, and, by the process of adhesion to it and of ulceration into its cavity, established a communication, it was still a desideratum to ascertain what portion of the bowels was thus affected. In ten days this orifice healed, and continued so till April 1815, a period of three months. At the expiration of this time another inflammation about the orifice took place, followed by suppuration and ulceration; feces were again discharged for ten days along with the pus. The parts then healed, and continued so till February 1817. During this interval the health became sufficiently good to induce him to consider himself exempt from the chance of similar attacks; but these hopes were destroyed, about the middle of February, by a recurrence of the inflammation in the thigh, with discharge of feces.

Between this date and the following August, four fresh attacks supervened; after that, he suffered from them every three or four weeks till December. It is now necessary to observe, that, in the recent recurrences of the disease, the old orifice, which had been situated not far from the anterior superior spinous process of the ilium, remained cicatrized, and a new one formed nearer the pubis. Also, that the inflammation which had been the percursor in every instance of the discharge of feces, was not attended latterly with suppuration, but that the integuments affected by it, being protruded by the accumulation of feces, burst and gave vent to them. The inflammation then subsided, and the part healed. It was therefore apparent that the feces occasioned the inflammation in the groin. The inflammation had, during this time, extended itself into the contiguous parts, and, amongst others, affected the inner side of the thigh, two-thirds of the way down to the knee.

The sequel of the case, as communicated to me by Dr. Gebhard, is as follows:—In December 1817, he first saw it. The patient was then labouring under a violent symptomatic fever: the most rigid antiphlogistic plan was pursued, such as bleeding, low diet, refrigerants, &c. By these means he was relieved from the constitutional affection. The subsequent exhibition of bark seemed to strengthen him, and to diminish the intensity of succeeding paroxysms, which took place in connexion with the bursting of feces through his thigh every two or three weeks till his death.

In February 1820, he became anasarcous, and continued so until the middle of July: he was then seized with a sore throat, which, in its sensations of pain, was extended along the œsophagus into the stomach. The sensibility of those parts became so highly excited, that the swallowing of such bland fluids as flaxseed tea, milk and water, &c. was excruciating. Examination showed the pharynx and palate inflamed, but no swelling of the tonsils. Gargles being ineffectually exhibited, the application of a blister on the throat relieved him. The dropsical affection began to disappear simultaneously with the commencement of the sore throat; and, by the time the latter was cured, the former had subsided entirely. A diarrhœa had attended the dropsical affection from its beginning; his health sunk apace under its influence, notwithstanding the continuance of a good appetite. The exhibition of medicines suppressed the discharge *per anum*, but produced no effect on that through the artificial opening. Wasted by this continual drain from the alimentary canal, he at length died on August 15th, 1820.

*Examination post mortem.*—The appearance of the abdominal viscera, generally healthy. The psoas magnus and iliacus internus muscle, which were considered to be the seat of the primary affection, had lost entirely their muscular fibres, were diminished in size, somewhat indurated, and converted into a ligamentous-like mass. The head of the colon, besides being bound down to the right iliac region by the usual reflection of peritoneum, had contracted an extensive adhesion to the contiguous muscles. The peritoneum at the part alluded to seemed unaffected by disease. It is known that the colon, at its head, is covered only in two-thirds of its circumference, and that anteriorly, by the peritoneum; the rest of this portion of the gut being in contact with the iliacus internus muscle, and connected to it, in health, by loose cellular substance: it is the latter part that I allude to, as having united itself to the iliacus muscle, by a preternatural adhesion, which was strong and so compact as to produce a continuity of substance with the muscle itself. At this place, about an inch and a half above the valve of the colon, two orifices existed through the parietes of the gut, each large enough to admit a finger: these orifices communicated with one fistula of the same size, in the centre of the iliacus internus. The fistula passed out of the abdomen, continuing in the centre of the muscle, till it had got beyond Poupart's ligament; it then became superficial, and terminated in the orifice of the groin, so often alluded to. A prosecution of the dissection exhibited another fistula arising from the lower termination of the first, and extending downwards six inches, parallel, or nearly so, with the femoral vessels. It was not known whether this fistula had arisen at the commencement of the disease, or from the aggravation of the local inflammation which took place about August 1817: its connexion with the other was subsequently demonstrated, by the ability of the patient to discharge feces from the external orifice, by pressing along the course of the adductor muscles from below upward. The femoral artery and vein were imbedded, as far as the place where they perforate the tendon of the triceps adductor, in a ligamentous sheath, which, I presume, had been fabricated in order to protect them from the extension of the disease in their vicinity. The parietes of each fistula were so thick and perfect, that they might have been, with ordinary facility, dissected from the contiguous parts, and preserved in their membranous form. The vertebræ of the loins were in a healthy condition.

This case I believe to have been unusual, in regard to the communication established between the abscess situated in the iliacus internus and psoas magnus muscle, and the cavity of the colon; and it may, perhaps, prove serviceable, by calling, in similar cases, the attention of the practitioner to the cause of a series of symptoms which embarrassed exceedingly all the medical gentlemen who were consulted about it. It is also a good example of the species of lumbar abscess, which, in the language of Mr. Abernethy, proceeds from phlegmonoid inflammation in the part. The circumstance is familiar to most surgeons that there are two species of lumbar abscess: the one preceded by pain, tumefaction, increased heat, throbbing, and a hurried circulation of blood through the loins, and a secretion of coagulating lymph, by which the parts are united to each other, and the boundaries of the abscess circumscribed; constituting, by all these symptoms, common inflammation. The other species belongs to chronic abscesses, in which collections of matter take place without any evident act of inflammation. In these latter, the surrounding parts remain in a great degree unaffected by the diseased action: the purulent discharge commences from very small beginnings, increases gradually, accumulates indefinitely; its boundaries are soft and unattended with thickening, affording but little impediment to its gravitation; and the matter, therefore, passes from one part to another, appearing sometimes in the groin, sometimes just above the knee, and occasionally in the perineum, giving occasion, by this change of place, to the distinction which Mr. Hunter has made between abscess *in a part, and of a part.* This last form of the disease, supposed to depend on a scrofulous habit, has its peculiar mode of treatment, which is very satisfactorily illustrated in Mr. Abernethy's Surgical Observations: but the former, differing essentially from it, and partaking largely of the attributes of common inflammation, has its remedial indications accordingly. The case just recounted seems to me, from the primary symptoms, and from the alteration of structure in the parts affected, to have been one of phlegmonoid inflammation; and would have been cured by the treatment, had not the diseased action extended itself through the parietes of the colon, and by that means produced a constant evacuation of feces into the cavity of the abscess, which eventuated in incurable fistula and artificial anus.—(*Philadelphia Journal of the Medical and Physical Sciences.* Edited by Dr. CHAPMAN. No. II.)

